# VANCOMYCIN- AND CHLORHEXIDINE DIGLUCONATE-IMPREGNATED DRESSING FOR THE PREVENTION OF ORTHOPEDIC SURGICAL SITE INFECTIONS: A PILOT STUDY USING A LOW-COST PROCEDURE

**DOI:** 10.1590/1413-785220253305e286896

**Published:** 2025-09-22

**Authors:** Paula Setti Campelo, Arthur Peruzzo Maziero, Marília França Madeira Manfrinato, Leticia Ramos Dantas, Paula Hansen Suss, Jamil Faissal Soni, Felipe Francisco Tuon

**Affiliations:** 1 Pontificia Universidade Catolica do Parana, Faculdade de Medicina, Hospital Universitario Cajuru, Curitiba, PR, Brazil.; 2 Pontificia Universidade Catolica do Parana, Faculdade de Medicina, Laboratorio de Doencas Infecciosas Emergentes, Curitiba, PR, Brazil.

**Keywords:** Dressing, Antibiotics, Trauma, Vancomycin, Chlorhexidine, Curativo, Antibióticos, Trauma, Vancomicina, Clorexidina

## Abstract

**Objective:**

This study introduces a novel approach by proposing a dressing impregnated with vancomycin and chlorhexidine for postoperative care, specifically targeting patients undergoing orthopedic surgeries.

**Methods:**

The research design involved a pilot study with a randomized control group, aiming to evaluate the efficacy and safety of vancomycin with a chlorhexidine dressing (Evidence Level I). Participants received vancomycin and CHG impregnated dressing or non-impregnated dressing during the period from March 2023 to October 2023 in trauma-related surgery in the lower limbs. The sample size was based on convenience, considering the pilot study; even though it was randomized and blinded, this study was not a clinical trial.

**Results:**

A total of 12 patients used an impregnated dressing, and nine used the same dressing without impregnation. Despite limitations, including the small sample size and single-center study location, our findings demonstrate the safety of the impregnated dressing in trauma surgeries, indicating potential applicability in broader surgical contexts.

**Conclusion:**

In conclusion, this study contributes to the discourse on preventive strategies for SSIs, presenting a pioneering approach with the use of vancomycin and chlorhexidine-impregnated dressings. Future research endeavors, incorporating larger-scale studies and addressing study limitations, are crucial for advancing the understanding and implementation of effective postoperative care strategies. ***Level of Evidence I; Case-control study.***

## RESUMO

**Objetivo:**

Este estudo introduz uma nova abordagem ao propor um curativo impregnado com vancomicina e clorexidina para cuidados pós-operatórios, visando especificamente pacientes submetidos a cirurgias ortopédicas.

**Métodos:**

O desenho da pesquisa envolveu um estudo piloto com grupo controle randomizado, com o objetivo de avaliar a eficácia e segurança do curativo de vancomicina com clorexidina (nível de evidência I). Os participantes receberam curativo impregnado de vancomicina e CHG ou curativo não impregnado durante o período de março de 2023 a outubro de 2023 em cirurgia relacionada a trauma em membros inferiores. O tamanho da amostra foi baseado em conveniência considerando o estudo piloto, mesmo sendo randomizado e cego, este estudo não foi um ensaio clínico.

**Resultados:**

12 pacientes utilizaram curativo impregnado e 9 utilizaram o mesmo curativo sem impregnação. Apesar das limitações, incluindo o pequeno tamanho da amostra e a localização do estudo em um único centro, nossos achados demonstram a segurança do curativo impregnado em cirurgias de trauma, indicando potencial aplicabilidade em contextos cirúrgicos mais amplos.

**Conclusão:**

Concluindo, este estudo contribui para o discurso sobre estratégias preventivas para ISC, apresentando abordagem pioneira com o curativo impregnado de vancomicina e clorexidina. Esforços de pesquisas futuras, incorporando estudos em maior escala e abordando as limitações dos estudos, são cruciais para avançar na compreensão e implementação de estratégias eficazes de cuidados pós-operatórios. ***Nível de Evidência I; Estudo de caso-controle.***

## INTRODUCTION

Advancements in medical technology have expanded surgical options, enabling more specific procedures across various medical domains worldwide. Despite these advancements, the management of surgical incisions remains a significant concern, irrespective of the surgical category or location.^1^ Over the past decades, there has been a growing emphasis on postoperative care, exemplified by the emergence of the Enhanced Recovery After Surgery (ERAS) protocol, which advocates interventions aimed at enhancing patient recovery and reducing postoperative complications.^2^ However, recommendations regarding the selection of dressings for postoperative surgical incision care, despite surgical site infection (SSI) being a common nosocomial infection, are notably lacking.

SSI affects up to 23.6 per 100 surgical procedures, with considerable implications for patients and healthcare systems.^3^ These infections are associated with increased morbidity, mortality, and treatment costs, resulting in an additional postoperative period of 7 to 11 days and an elevated risk of death by 2 to 11 times compared to postoperative patients without SSI. In the field of orthopedic surgery, SSIs are recognized as a crucial risk factor for complications and unfavorable outcomes.^4–7^ The healthcare costs associated with these complications exceed $1.6 billion in the United States alone.^8^ Among the resources for preventing SSIs in orthopedics, various types of dressings can be employed to preserve the physiological wound healing process and prevent potential infections, improving early discharge. These dressings can be broadly classified into four categories: traditional or passive, skin substitutes, interactive materials, and bioactive dressings.^9^ Bioactive dressings, in particular, play a significant role by providing active materials, such as antibiotics, to the wound site.^10^ These materials can be impregnated in structures like nanofibers, wafers, foams, sponges, hydrogels, membranes, and films, each with advantages to enhance their biological activity.^11^ Moreover, the widespread use of antibiotics has led to the emergence of resistant strains, and postoperative bioactive dressings emerge as a potential solution by minimizing systemic effects through local release, reducing the likelihood of microbial resistance.^12–17^ Additionally, surgical site infections can occur post-discharge, where dressings offer the convenience and superior adherence to treatment.^18^

Different dressing structures have distinct advantages and disadvantages. Sponges provide thermal insulation, maintain a moist environment at the wound site, and exhibit high porosity; however, they are mechanically fragile.^19^ On the other hand, hydrogels can store a significant amount of water within their 3D polymeric network, facilitating a moist environment for healing, but require a secondary dressing due to their mechanically fragile properties.^20^ Hydrocolloids, as non-adherent and painless dressings, can be easily removed by saline or sterile water but may be cytotoxic, have an unpleasant odor, and maintain an acidic pH at the wound site. Films effectively block the flow of liquids and bacteria while allowing free passage of oxygen and water vapor.^21^ Due to their flexible, lightweight, and low water absorption characteristics, films are suitable for treating delicate skin and superficial wounds with low exudation. However, they can be challenging to handle, adhere to the wound bed, and cause accumulation of exudate. Membranes are known to act as physical barriers, replicate the three-dimensional architecture of the native extracellular matrix, and ensure cell proliferation, gas exchange, and nutrient supply. They are a semipermeable biomaterial, similar to films, but with a higher water absorption capacity. However, their use is limited by the materials and solvents used in their production.^22^

In the context of various dressing types, a Cochrane review conducted in 2011 and subsequently updated (2022) examined different dressing strategies, including studies of wounds without dressings, but found insufficient evidence to draw a reliable conclusion about the superiority of any one dressing.^11^ This was due to the inadequate quality of available evidence, with most studies being limited in size and considered susceptible to systematic errors. Therefore, the authors recommend further research to advance this field.

Among the drugs producing the desired antimicrobial effect in dressings, chlorhexidine gluconate (CHG), an antiseptic that has demonstrated efficacy in reducing the risk of catheter-associated blood infections, is noteworthy.^23^ However, despite its promising use, there is still a gap in scientific knowledge regarding the use of dressings impregnated with CHG in preventing surgical site infections and reducing bacterial load.

Other drugs with similar effects include antibiotics, among which vancomycin stands out due to its widespread use in clinical therapies and its antimicrobial potential against resistant Gram-positive pathogens present in the early stages of infection. However, maintaining therapeutic systemic levels of this antibiotic can be challenging and lead to adverse effects such as nephrotoxicity. Local vancomycin release avoids systemic side effects, with topical use already showing promise in the treatment of osteomyelitis, for example. Thus, this research proposes a dressing impregnated with vancomycin and CHG for postoperative care of patients undergoing orthopedic surgeries to prevent surgical site infections by delivering antimicrobials directly to the affected site.

## METHODS

### Dressing development

The impregnated dressing was developed based on a 20% chlorhexidine digluconate solution, diluted with vancomycin. The solution contained 5% chlorhexidine and 125mg/L vancomycin. Following the formulation of the solution, a 300 gsm filter paper was immersed for a sufficient absorption period and subsequently subjected to freeze-drying, followed by ethylene oxide sterilization. Preliminary microbiological tests for the dressing were conducted using the Kirby-Bauer method.^24^ For this, 6 mm diameter disks were created and placed on a Muller-Hinton agar plate previously inoculated with *Staphylococcus aureus* ATCC® 25923.^25^ The resulting inhibition zone was compared with a disk made of the same material but without impregnation.^26^ The impregnated dressings were assessed for efficacy over 7 days on human skin. Volunteers received the dressing in the form of 6 mm diameter disks, which were placed on healthy skin for a duration of 7 days. After this period, the disks were removed from the skin and placed on a Muller-Hinton plate previously inoculated with *S. aureus* ATCC® 25923. The inhibition zone was compared with a disk made of the same material but without impregnation.

### Study design and sample size

This is a pilot study evaluating the use of vancomycin and CHG dressings for immediate wound coverage (post-operative) for seven days to prevent surgical site infections. It was a controlled and randomized study (non-impregnated dressing) involving a total of 23 participants who underwent elective and emergency fracture repair surgery at a University Hospital (CAAE 68749223.9.0000.0020). Participants received vancomycin and CHG impregnated dressing or non-impregnated dressing during the period of March 2023 to October 2023. The sample size was based on convenience, considering the pilot study; even though it was randomized and blinded, this study was not a clinical trial.

### Ethical aspects

The study was submitted and approved by the Research Ethics Committee. Dressings impregnated with vancomycin and CHG were produced at the LEID (Laboratory of Emerging Infectious Diseases) of the Pontifical Catholic University of Paraná. The proportions and properties of the dressings used in this study cannot be disclosed at this time, as they are protected by patent law (Law No. 9.279, May 14, 1996, Brazil). The control dressing consisted of the same material without impregnation. All participants must sign the Informed Consent Form, and interventions will only occur after the document has been signed.

### Inclusion and exclusion criteria

The inclusion criteria are adult patients (18 years or older) who are already scheduled for the surgical procedure and provide informed consent to participate in the study. Exclusion criteria were patients allergic to vancomycin or CHG, with a known history of allergy to any of the drugs, recent infectious process, or immunocompromised.

### Randomization

Randomization was done through sealed envelopes indicating the type of dressing to be used. The patient and the surgeon were blinded, with only the person responsible for the dressing production aware of the dressing type. The statistical analysis was blinded, and the groups were recognized after the final analysis.

### Intervention and outcome

On the surgery day (day 0), the assigned dressing was applied to the surgical site intraoperatively after the sutures were placed. A photograph of the sutured surgical incision was taken with pre-calibrated rulers placed adjacent to the surgical site for subsequent 2D morphometric analysis. Gauze will then be placed over the dressing, followed by a bandage over the entire surgical area. Participants were instructed not to disturb the dressings for the first 24 h. The first dressing change occurred 24 hours after admission (day 1) in the hospital setting, during the hospitalization. The second change occurred 48 hours post-procedure (day 2), after discharge, and was to be performed by the patient, who was instructed accordingly.

The next assessment took place after seven days (day 7) post-surgery in an outpatient setting at the same hospital. 2D morphometric analysis of the wound was conducted, with pre-calibrated rulers placed adjacent to the surgical site for a new photograph. Additionally, the doctor executed the Bluebelle Wound Healing Questionnaire (WHQ) and collected sociodemographic data for qualitative analysis of the surgical wound and the healing process. Surgical site infection was the primary outcome and was evaluated until day 30. The definition of SSI was based on CDC (Center for Diseases Control) criteria, which included: 1) occurs within the first 30 days after surgery and involves only the skin and subcutaneous tissue; 2) purulent drainage from the superficial incision OR positive culture of secretion or tissue from the superficial incision, obtained aseptically (cultures collected by swab are not considered); 3) the superficial incision is intentionally opened by the surgeon in the presence of at least one of the following signs or symptoms: pain, increased sensitivity, local edema, hyperemia, or warmth, UNLESS the culture is negative. Safety was evaluated as adverse events associated with the dressing, including rash, pain, infection, bleeding, necrosis, and dehiscence. The follow-up was 30 days.

### Statistical analysis

Student's t-test compared continuous variables and was expressed in absolute numbers or percentages. The categorical variables were analyzed by chi-square or Fisher test and expressed as absolute numbers or percentages. Statistical difference was considered when p <0.05. SPSS v23 was used for statistical analysis (IBM, Armonk NY).

## RESULTS

**Figure 1:**
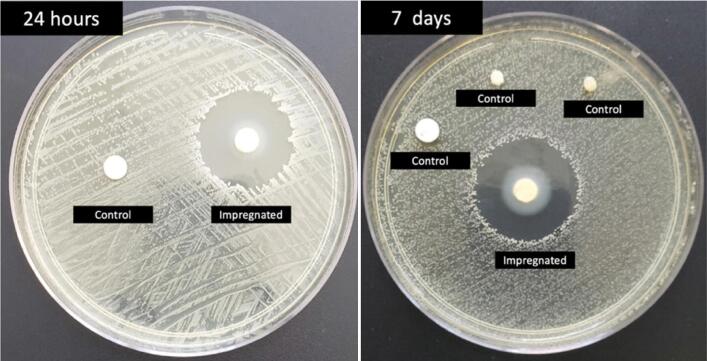
Disk testing of vancomycin-CHG impregnated dressing 24 h after impregnation and 7 days after continuous skin contact.

Microbiological tests demonstrated that the dressings exhibited significant antimicrobial activity against S. aureus, a pivotal bacterium in SSI (Figure 1). In the 7-day validation, the dressings maintained their activity. This test is crucial for defining the duration of dressing use, preventing unnecessary applications due to activity loss, and potentially extending the usage time for patients lacking optimal wound care conditions — a common scenario in developing countries. The 24-hour inhibition halo presented a median of 28+/-2.5 mm (n = 10). The inhibition halo was absent in all control dressings (n=10).

**Figure 2:**
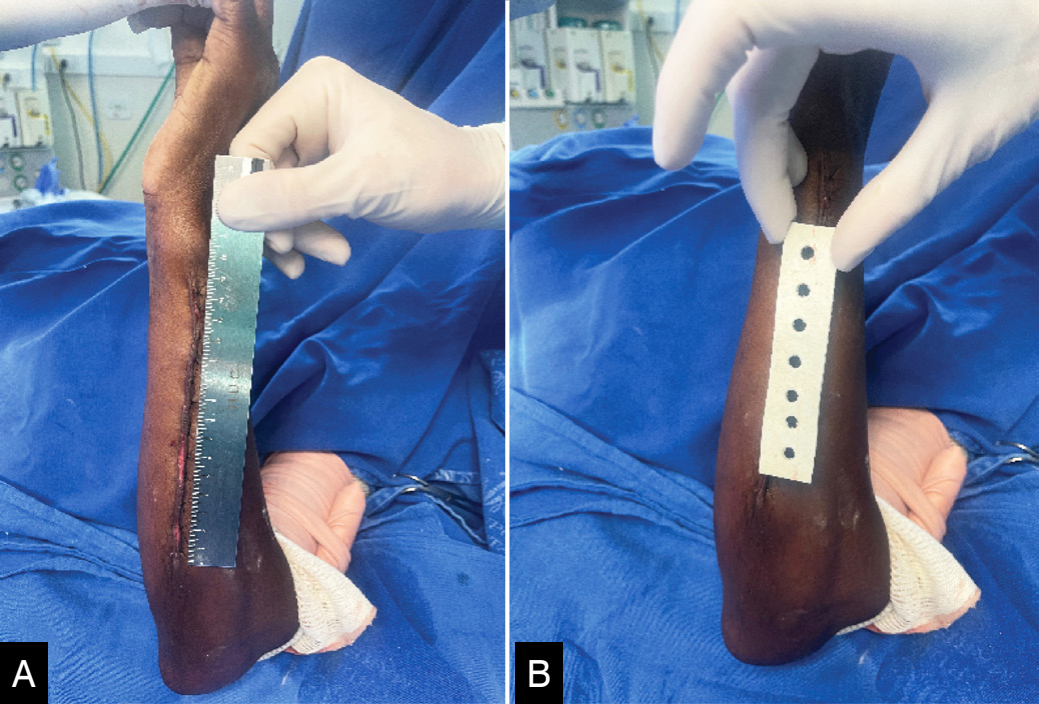
Surgical wound before (A) and after (B) vancomycin-CHG impregnated dressing.

A total of 23 randomized patients were enrolled, with 2 excluded due to non-infectious compartment syndrome requiring re-intervention. Consequently, 21 patients were included for the application of dressing. Among the 21 included patients, 12 received the impregnated dressing (57.14%), and 9 received the control dressing (52.38%). The median age was 40 years (IQR 25-75% 28-44). Comorbidities were observed in 28.57% of patients, including three with systemic arterial hypertension and two with diabetes mellitus, one of whom had both comorbidities. Smoking, a risk factor for not healing, was present in 19.04% of patients. Regarding gender, 90.47% (19/21) were male. The data for group comparison are presented in Table 1. Considering the small number of patients in the pilot study, no statistical difference was observed between the groups. Only one case of SSI occurred, which was identified in the control group. Therefore, it is possible to consider that the impregnated dressing is equivalent to the control group, demonstrating its safe use. In Figure 2, the appearance of the dressing in the surgical area before it is covered with a transparent film can be observed.

**Table 1:** Clinical and outcome data of patients under vancomycin-chlorhexidine impregnated dressing and the control group. SAH – Systemic arterial hypertension; DM – diabetes mellitus; SD – Standard deviation.

	Impregnated dressing (n=12)	%	Control (n=9)	%	Total	%	P value
Age (median/IQR 25-75%)	40 (30-53)		39 (28-41)		40 (28-44)		0.222
Male sex	11	91.66	8	88.88	19	90.47	0.686
Comobidities			1	11.11	1	4.76	0.324
SAH	3	25	1	11.11	4	19.04	
DM	2	16.67	1	11.11	3	14.28	
Smoking	2	16.67	2	22.22	4	19.04	0.353
Trauma							0.523
Lower limbs	9	75	6	66.67	15	71.42	
Upper limbs	3	25	3	33.33	6	28.57	
Infection	0	0	1	11.11	1	4.76	0.429

## DISCUSSION

Antimicrobial dressings are routinely applied in the care of postoperative incisions, and the development of these dressings has garnered significant attention to meet the growing demands for wound protection against Surgical Site Infections (SSIs) and wound healing promotion.^27^ Aligned with the global purpose of developing dressings with these attributes, our study, albeit in a pilot phase, enriches this discussion by presenting a potential strategy in the form of a dressing impregnated with vancomycin and CHG. This specific antimicrobial combination has not undergone prior investigations, highlighting the pioneering nature of this research in SSI prevention. Regarding CHG, its efficacy in reducing MRSA contamination in surgical wounds has been previously demonstrated in a porcine model, showing significant antimicrobial activity and highlighting the potential of CHG dressings against MRSA.^28^ Furthermore, meta-analyses support this by stating that dressings impregnated with CHG significantly reduce infections related to central venous catheters, with rare adverse effects.^29^ As for vancomycin, studies such as Kalalinia et al. (2021) presented vancomycin-loaded hybrid nanofibers, demonstrating a significant absence of cytotoxicity and effective inhibition of bacterial growth, highlighting the safe and effective use of vancomycin in this topical application.^30^ However, the effectiveness of the dressing proposed by our pilot study cannot be compared with those described at this time due to the small sample size.

Therefore, given the lack of consensus regarding postoperative wound care, leaving the choice of dressing to the individual discretion of the physician, coupled with the limitations and uncertainties raised by previous studies, the urgent need for advancing research in this domain is emphasized.^28^ Given the increasing relevance of the topic, the lack of substantial data, and the pilot nature of this study, we foresee, in line with the envisioned approach in previous research, the conception and execution of a randomized, double-blind, large-scale study with homogeneous sampling addressing the new dual-action antimicrobial dressing impregnated with vancomycin and CHG in operative wounds, aiming to achieve both statistical and clinical significance. Despite the small number of patients in our study, which precluded a comparative evaluation of infection rates due to the low incidence of this type of trauma at this time, the new dressing proved to be safe in trauma surgeries, indicating promising applicability in other surgeries.

Regarding the study limitations, this is a pilot study rather than a large-scale clinical trial, which limits the ability to draw robust conclusions about the effectiveness of vancomycin and CHG dressings. Additionally, undisclosed properties of the dressings hinder replication in studies at other centers now. Conducted at a single University Hospital in Curitiba, PR, the study's findings may not be universally applicable. Therefore, future research addressing these limitations can enhance the overall assessment of the dressing's effectiveness in preventing Surgical Site Infections.

This study aimed to evaluate the efficacy and safety of operative wound dressings impregnated with vancomycin and CHG, representing an innovative approach to preventing SSI and highlighting the lack of prior research on this specific antimicrobial combination. We grounded our research in previous studies by considering the individual efficacy of CHG and vancomycin. However, the small number of patients did not allow for a comparative evaluation of the infection rate due to the low incidence of this type of trauma. Finally, based on the absence of adverse effects in the sample, our study demonstrated that this new dressing is safe for use in trauma surgeries, with potential applicability in other surgical procedures.

## References

[1] Sartelli M, Labricciosa FM, Coccolini F, Coimbra R, Abu-Zidan FM, Ansaloni L (2022). It is time to define an organizational model for the prevention and management of infections along the surgical pathway: a worldwide cross-sectional survey. World J Emerg Surg.

[2] Tao J, Yan Z, Bai G, Zhang H, Li J (2023). Enhanced Recovery after Surgery Rehabilitation Protocol in the Perioperative Period of Orthopedics: A Systematic Review. J Pers Med.

[3] Birhanu A, Amare HH, G/Mariam M, Girma T, Tadesse M, Assefa DG (2022). Magnitude of surgical site infection and determinant factors among postoperative patients, A cross sectional study. Ann Med Surg.

[4] Tuon FF, Suss PH, Telles JP, Dantas LR, Borges NH, Ribeiro VST (2023). Antimicrobial Treatment of *Staphylococcus aureus* Biofilms. Antibiotics.

[5] Ribeiro VST, Cieslinski J, Bertol J, Schumacher AL, Telles JP, Tuon FF (2021). Detection of Microorganisms in Clinical Sonicated Orthopedic Devices Using Conventional Culture and qPCR. Rev Bras Ortop (Sao Paulo).

[6] Tuon FF, Dantas LR, Suss PH, Tasca Ribeiro VS (2022). Pathogenesis of the *Pseudomonas aeruginosa* Biofilm: A Review. Pathogens.

[7] da Rocha LGDO, Ribeiro VST, de Andrade AP, Gonçalves GA, Kraft L, Cieslinski J (2022). Evaluation of Staphylococcus aureus and Candida albicans biofilms adherence to PEEK and titanium-alloy prosthetic spine devices. Eur J Orthop Surg Traumatol.

[8] Worldwide Antimicrobial Resistance National/International Network Group (WARNING) Collaborators (2023). Ten golden rules for optimal antibiotic use in hospital settings: the WARNING call to action. World J Emerg Surg.

[9] Motelica L, Vasile BS, Ficai A, Surdu AV, Ficai D, Oprea OC (2023). Antibacterial Activity of Zinc Oxide Nanoparticles Loaded with Essential Oils. Pharmaceutics.

[10] Aldakheel FM, Mohsen D, El Sayed MM, Fagir MH, El Dein DK (2023). Employing of Curcumin-Silver Nanoparticle-Incorporated Sodium Alginate-Co-Acacia Gum Film Hydrogels for Wound Dressing. Gels.

[11] Ribeiro CT, Dias FA, Fregonezi GA (2022). Hydrogel dressings for venous leg ulcers. Cochrane Database Syst Rev.

[12] Arend L, Bergamo R, Rocha FB, Bail L, Ito C, Baura VA (2023). Dissemination of NDM-producing bacteria in Southern Brazil. Diagn Microbiol Infect Dis.

[13] Chaiben V, Yamada CH, Telles JP, de Andrade AP, Arend L, Ribeiro VST (2022). A carbapenem-resistant Acinetobacter baumannii outbreak associated with a polymyxin shortage during the COVID pandemic: an in vitro and biofilm analysis of synergy between meropenem, gentamicin and sulbactam. J Antimicrob Chemother.

[14] Telles JP, Yamada CH, Dario TM, Miranda AN, Pacheco A, Tuon FF (2022). Impact of an antimicrobial stewardship program in a COVID-19 reference hospital according to the AWaRe classification. Am J Infect Control.

[15] Ito CAS, Bail L, Arend LNVS, Nogueira KDS, Tuon FF (2021). The activity of ceftazidime/avibactam against carbapenem-resistant *Pseudomonas aeruginosa*. Infect Dis.

[16] Bail L, Ito CAS, Arend LNVS, Pilonetto M, Nogueira KDS, Tuon FF (2021). Distribution of genes encoding 16S rRNA methyltransferase in plazomicin-nonsusceptible carbapenemase-producing Enterobacterales in Brazil. Diagn Microbiol Infect Dis.

[17] Zequinão T, Telles JP, Gasparetto J, Tuon FF (2020). Carbapenem stewardship with ertapenem and antimicrobial resistance-a scoping review. Rev Soc Bras Med Trop.

[18] Chiquin CA, Silva JH, Ciruelos MJ, Lemes MC, Penteado-Filho SR, Tuon FF (2009). Postdischarge surveillance system for nontuberculous mycobacterial infection at a Brazilian regional referral hospital after an outbreak. Infect Control Hosp Epidemiol.

[19] Oprica GM, Panaitescu DM, Usurelu CD, Vlasceanu GM, Stanescu PO, Lixandru BE (2023). Nanocellulose Sponges Containing Antibacterial Basil Extract. Int J Mol Sci.

[20] Suneetha M, Won SY, Zo SM, Han SS (2023). Fungal Carboxymethyl Chitosan-Impregnated Bacterial Cellulose Hydrogel as Wound-Dressing Agent. Gels.

[21] Awasthi A, Gulati M, Kumar B, Kaur J, Vishwas S, Khursheed R (2022). Recent Progress in Development of Dressings Used for Diabetic Wounds with Special Emphasis on Scaffolds. Biomed Res Int.

[22] Wali N, Wajid N, Shabbir A, Ali F, Shamim S, Abbas N (2024). Considerations for Lyophilized Human Amniotic Membrane Impregnated with Colistin and Silver Nanoparticles. Appl Biochem Biotechnol.

[23] Xu H, Hyun A, Mihala G, Rickard CM, Cooke ML, Lin F (2024). The effectiveness of dressings and securement devices to prevent central venous catheter-associated complications: A systematic review and meta-analysis. Int J Nurs Stud.

[24] de Andrade AP, Arend LNVS, Ribeiro VST, Tuon FF (2022). Resistance of clinical and environmental Acinetobacter baumannii against quaternary ammonium. Infect Control Hosp Epidemiol.

[25] Mendonca JR, Dantas LR, Tuon FF (2023). Activity of multipurpose contact lens solutions against Staphylococcus aureus, Pseudomonas aeruginosa, Serratia marcescens and Candida albicans biofilms. Ophthalmic Physiol Opt.

[26] Pedroni MA, Ribeiro VST, Cieslinski J, Lopes APA, Kraft L, Suss PH (2024). Different concentrations of vancomycin with gentamicin loaded PMMA to inhibit biofilm formation of Staphylococcus aureus and their implications. J Orthop Sci.

[27] Jiang N, Rao F, Xiao J, Yang J, Wang W, Li Z (2020). Evaluation of different surgical dressings in reducing postoperative surgical site infection of a closed wound: A network meta-analysis. Int J Surg.

[28] Mana TSC, Donskey C, Carty N, Perry L, Leaper D, Edmiston CE (2019). Preliminary analysis of the antimicrobial activity of a postoperative wound dressing containing chlorhexidine gluconate against methicillin-resistant Staphylococcus aureus in an in vivo porcine incisional wound model. Am J Infect Control.

[29] Buetti N, Rickard CM, Timsit JF (2022). Catheter dressings. Intensive Care Med.

[30] Kalalinia F, Taherzadeh Z, Jirofti N, Amiri N, Foroghinia N, Beheshti M (2021). Evaluation of wound healing efficiency of vancomycin-loaded electrospun chitosan/poly ethylene oxide nanofibers in full thickness wound model of rat. Int J Biol Macromol.

